# Genomic analysis of Nigerian indigenous chickens reveals their genetic diversity and adaptation to heat-stress

**DOI:** 10.1038/s41598-024-52569-4

**Published:** 2024-01-26

**Authors:** Mifta P. Rachman, Oladeji Bamidele, Tadelle Dessie, Jacqueline Smith, Olivier Hanotte, Almas A. Gheyas

**Affiliations:** 1 School of Biosciences, University of Nottingham, Nottingham, LE12 5RD UK; 2 African Chicken Genetic Gains (ACGG), Department of Animal Sciences, Obafemi Awolowo University, Ile Ife, 220282 Nigeria; 3 LiveGene-CTLGH, International Livestock Research Institute (ILRI), P.O. Box 5689, Addis Ababa, Ethiopia; 4 Centre for Tropical Livestock Genetics and Health (CTLGH), Roslin Institute, University of Edinburgh, Edinburgh, EH25 9RG UK; 5 School of Life Sciences, University of Nottingham, Nottingham, NG7 2RD UK

**Keywords:** Computational biology and bioinformatics, Evolution, Genetics, Zoology

## Abstract

Indigenous poultry breeds from Africa can survive in harsh tropical environments (such as long arid seasons, excessive rain and humidity, and extreme heat) and are resilient to disease challenges, but they are not productive compared to their commercial counterparts. Their adaptive characteristics are in response to natural selection or to artificial selection for production traits that have left selection signatures in the genome. Identifying these signatures of positive selection can provide insight into the genetic bases of tropical adaptations observed in indigenous poultry and thereby help to develop robust and high-performing breeds for extreme tropical climates. Here, we present the first large-scale whole-genome sequencing analysis of Nigerian indigenous chickens from different agro-climatic conditions, investigating their genetic diversity and adaptation to tropical hot climates (extreme arid and extreme humid conditions). The study shows a large extant genetic diversity but low level of population differentiation. Using different selection signature analyses, several candidate genes for adaptation were detected, especially in relation to thermotolerance and immune response (e.g., cytochrome P450 2B4-like, *TSHR*, *HSF1, CDC37*, *SFTPB, HIF3A, SLC44A2,* and *ILF3* genes). These results have important implications for conserving valuable genetic resources and breeding improvement of chickens for thermotolerance.

## Introduction

It is important to recognize the value of indigenous livestock populations from various geographic regions. These animals have adapted to their local agro-climatic conditions, making them important genetic resources for conservation efforts. By protecting these populations, we can help preserve their unique genetic traits and ensure the sustainability of our agricultural practices. Native tropical breeds are particularly crucial. As climate change and global warming are forcing many temperate regions to experience tropic-like conditions, such breeds may hold genetic solutions for climate resilience.

Nigeria is a tropical lowland country where poultry farming plays a crucial role in the economy and livelihood of local people. About 45% of the Nigerian population is involved in poultry production, mostly small or medium-scale farming, and Nigeria ranks second for its chicken population size (180 M birds) within Africa^1,2^. However, despite the importance of poultry farming for the country’s economy, over half of its chickens are still raised in extensive backyard farming systems. Moreover, about 80% of the chickens reared in backyard farming in Nigeria are represented by unimproved local breeds^3^. Being unimproved, they have poor productivity, but otherwise have very desirable qualities such as hardiness to thrive under harsh tropical environments, the ability to forage for food, the ability to hatch on their own and brood, and considerable tolerance to endemic disease challenges^4^. Besides this, their egg and meat products are preferred by local people^5^. These local chickens, commonly called Nigerian Indigenous Chickens (NICs), represent important genetic resources for the sustainable development of the poultry programme in Nigeria to cater for future needs arising from climate challenges and consumer demands.

**Figure 1:**
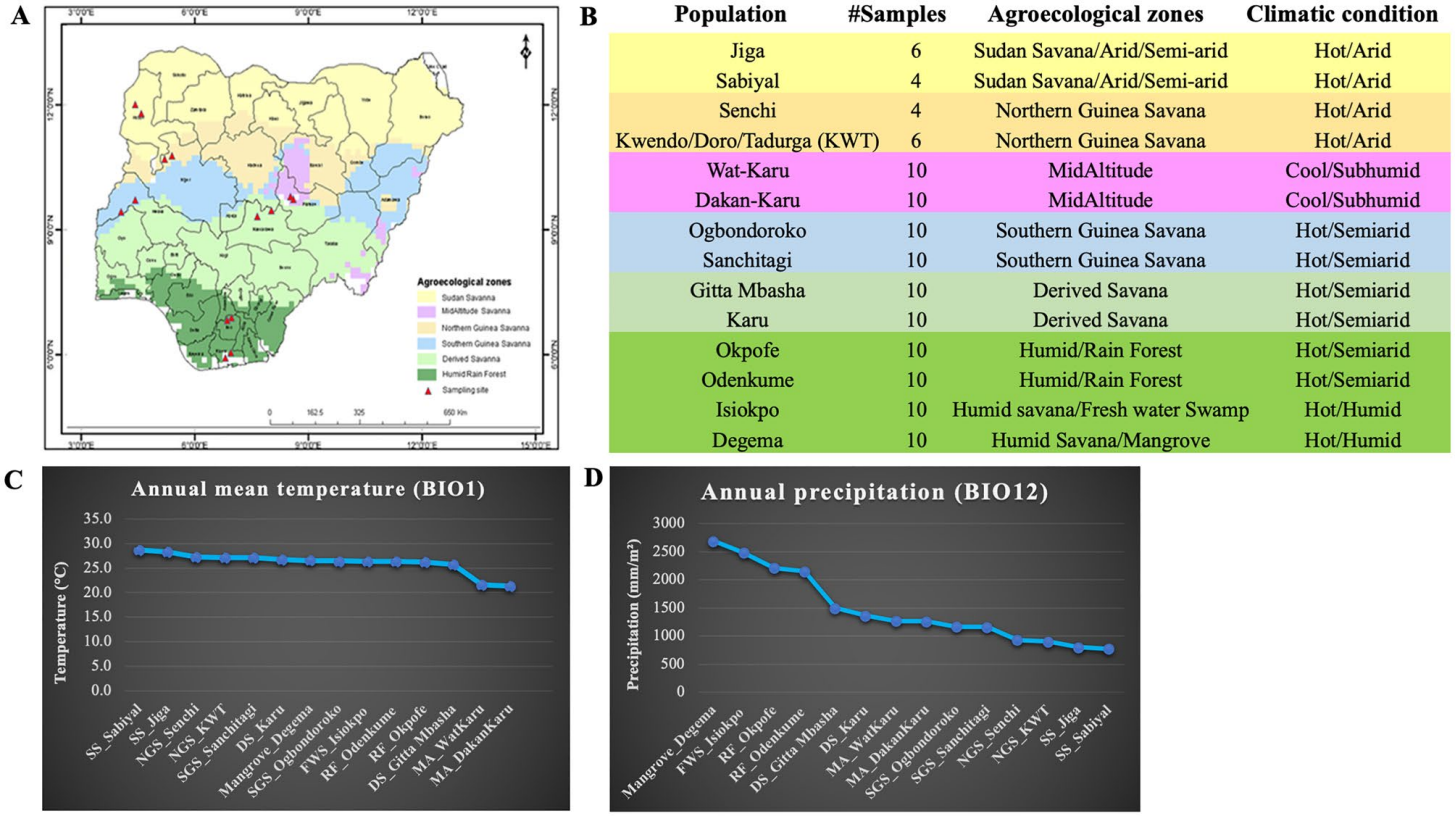
(**A**) Nigerian agro-ecological map showing sampling locations (figure modified from https://redd.unfccc.int/files/nigeria_national_frel_modified_revised__for_posting.pdf), (**B**) details of the studied chicken populations, (**C**) ordering of populations based on mean annual temperature, (**D**) ordering of populations based on mean annual precipitation. The means in (**C**) and (**D**) are based on 40 years of data (1960–2000) from the Worldclim database^11^.

The NICs surviving in varied Nigerian agro-climatic conditions offer an excellent opportunity to dissect tropical environmental adaptation, particularly thermotolerance in chickens, both under hot-humid and hot-arid conditions. Nigeria's landscape has been classified into several agroecological zones (AEZs). Transiting from a South to North direction, these include Mangrove Swamp and Coastal Vegetation, Freshwater Swamp Forest, Lowland Rain Forest, Derived Savanna, Guinea Savanna, Sudan Savanna, and Sahel Savanna^6^. In addition, there are a few mountainous areas found in the Jos Plateau, Adamawa, Taraba, and the Northern part of Cross River State (Fig. 1). Whilst most Nigerian geographic regions experience very high temperatures (except in the high plateaus), the climate varies from very wet conditions in the coastal South (annual rainfall > 3500 mm, temperature up to 32 °C) to extreme arid conditions in the Sahel region of the North-West and North-East (annual rainfall < 600 mm, temperature up to 41 °C)^7^. NICs can be classified into ecotypes in different ways. Based on geographical location, the NICs are classified into two major breeds or ecotypes: Fulani and Yoruba^8^. The Fulani ecotype is found in the Sahel and Guinea savanna, the cattle Kraals and Montane parts of northern Nigeria, whereas the Yoruba ecotype is located around the rainforest, swamps, and derived savanna areas^8^. Alternatively, agro-climatic regions can also be used to classify the NICs into potential ecotypes, such as mangrove, freshwater swamp forest, rainforest, derived savanna, Guinea savanna, Sudan savanna, and Sahel savanna^9^. The genetic characterisation of NICs from different ecotypes, particularly those based on agroecological zones, is crucial for conserving genetic and adaptive diversity and elucidating the molecular mechanisms of environmental adaptation. In particular, the prevalence of a very high temperature across most parts of the Nigerian landscape provides an excellent opportunity to investigate the genetic basis of heat stress adaption in general, and those specific to hot-humid and hot-arid conditions.

Until now, most of the genetic studies on NICs have been based either on mtDNA or microsatellite markers^10^. No study so far reports genetic and adaptive diversity based on whole-genome sequence (WGS) data. In this study, we examine the genetic diversity in NICs with WGS data from a large number of samples representing different agro-climatic zones. Using the dense genetic variants detected in this study, we investigate the signatures of positive natural selection in the NICs in response to heat stress in humid and arid conditions.

## Results

### Whole-genome sequencing shows a large within-population genetic diversity in NICs

In the present study, we sequenced and analysed 120 village chicken samples from 14 different populations (4–10 samples/population) which represent diverse AEZs across Nigeria (Fig. 1A,B). There is little variation in the mean annual temperature among these AEZs except in the mid-altitude region with a slightly lower temperature (Fig. 1C). However, the AEZs show a large variation in mean annual rainfall patterns (Fig. 1D). The genomes of 120 chickens were sequenced with 1.5 billion and 6 billion clean reads for each sample. The reads were then aligned to the chicken reference genome (GRCg6a) at an average mapping rate of 99% with a mean genomic coverage of ~ 64 × after mapping (Table S1). Using a joint analysis of all the samples, we detected over 17 M SNPs, of which ~ 11% (1.9 M) are novel. For downstream analysis, population wise SNPs were extracted which resulted in 8.9 million to 11.8 million SNPs per population (Table S1).

### NICs show low levels of population differentiation

**Figure 2:**
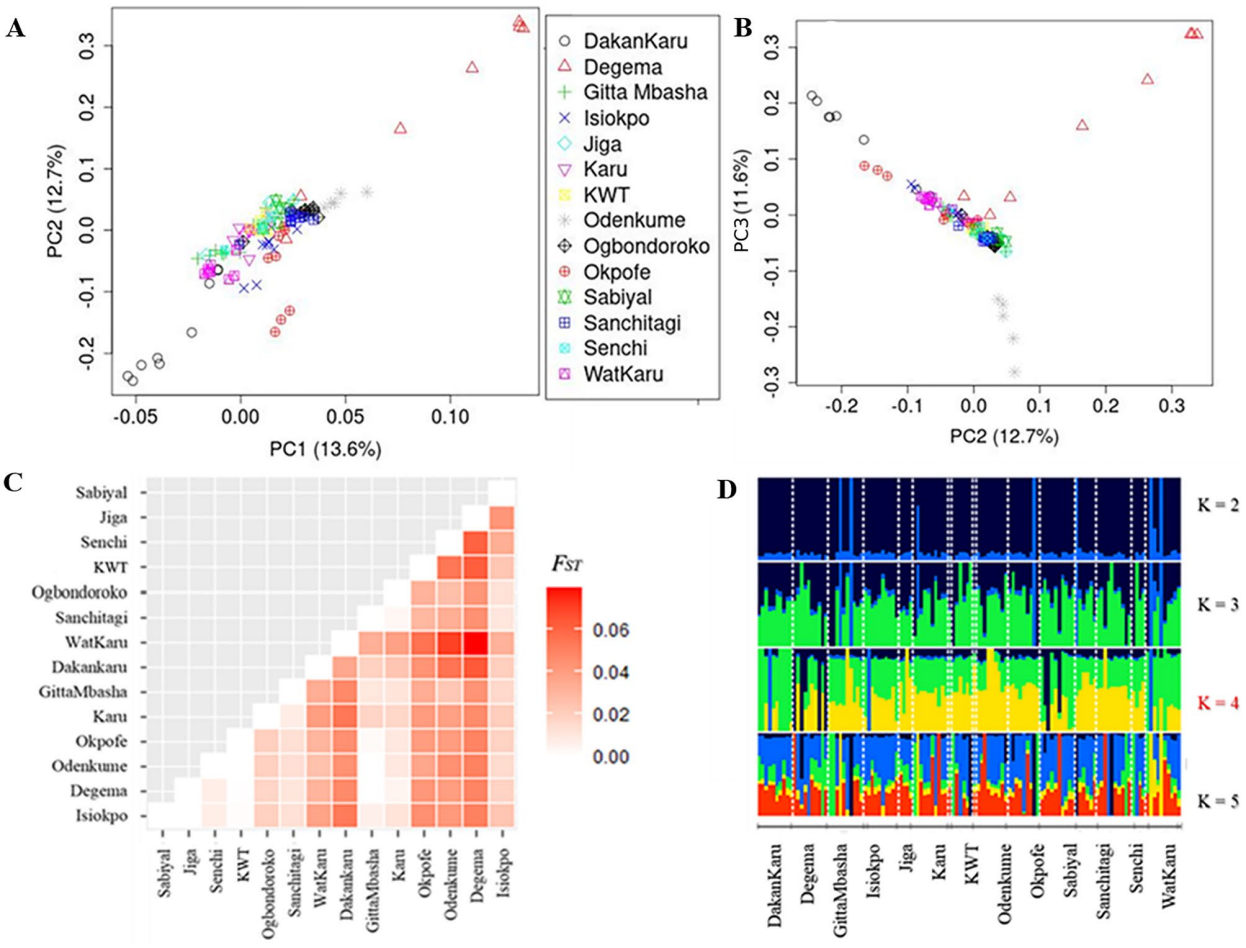
Population structure and genetic diversity: (**A**,**B**) PCA plots showing population structure, (**C**) heat map of pairwise *Fst* values, (**D**) admixture analysis (K = 4 is the best prediction).

Population structure and between-population differentiation were investigated using different approaches: Principal Component Analysis (PCA), admixture analysis, and *Fst* analysis*.* PCA plots show most populations clustered together except for those of Degema, DakanKaru and Odenkume (Fig. 2A,B). Pairwise *Fst* analyses show a generally low level of population differentiation (*Fst* < 0.05; indicating no or negligible differentiation), except in a few cases where moderate differentiation (0.05–0.08) was observed (Fig. 2C); these few cases involved the same populations (i.e., Degema, Dakan-Karu and Odenkume). Admixture analysis predicted contributions from four ancestral gene pools (Fig. 2D), but closer inspection indicates that it may have been due to the presence of some exotic birds in different populations. While most samples (at K = 4) show a mixture of two ancestral gene pools (shown by green and yellow colours), a few samples from different populations appear to have a completely different origin (shown either by black or blue colours).

### Genome-wide linkage disequilibrium (LD) structure confirms large genetic diversity

**Figure 3:**
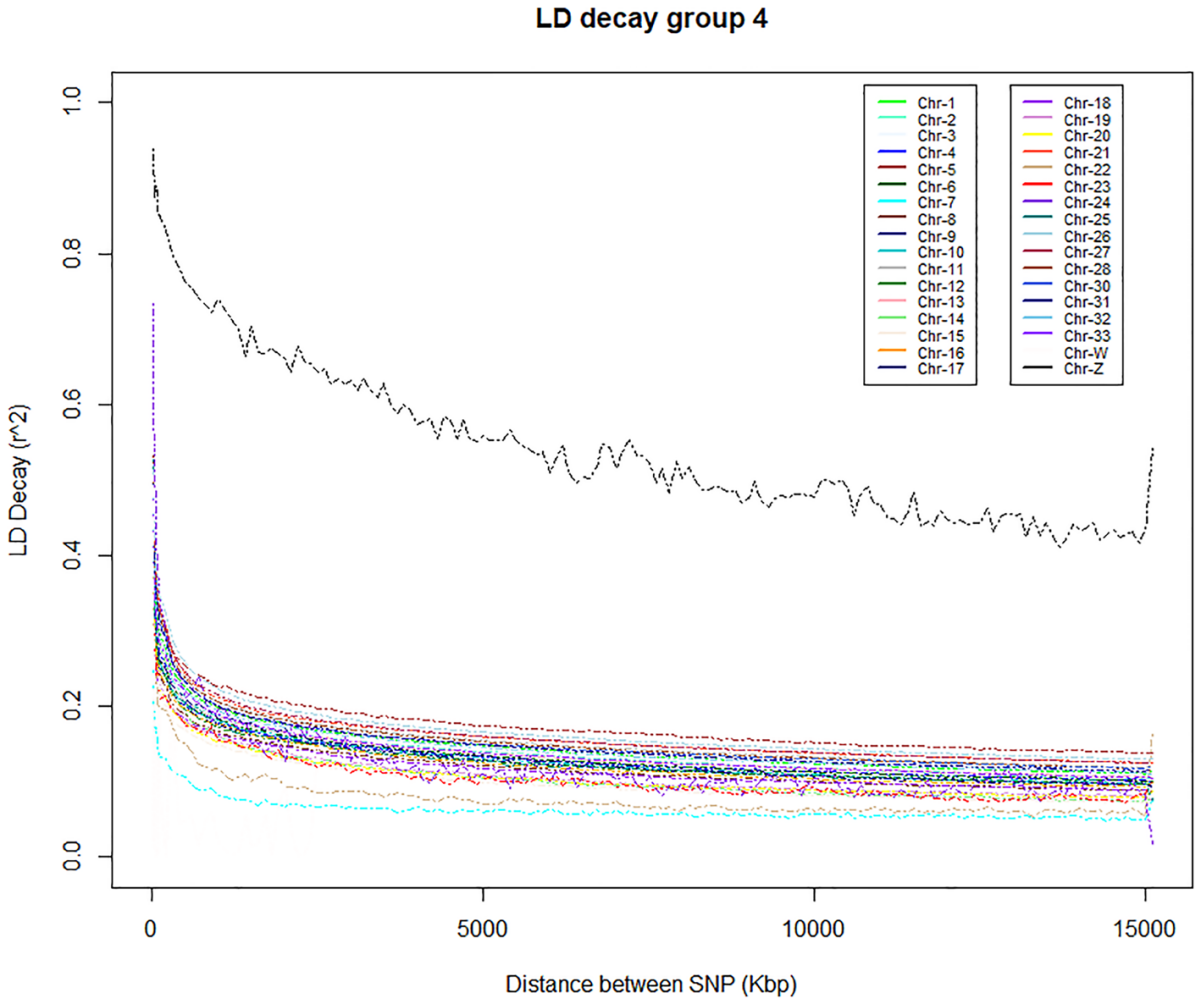
Chromosome-wise LD decay in a representative Nigerian chicken population groups.

LD decay analysis in our study shows that LD (*r*^2^) in NIC genomes drops rapidly with distance. The LD (*r*^2^) values ranged from 0.20 to 0.30 in 10 kbp distance for all chromosomes except for the Z-chromosome, which has the slowest LD decay rate. LD decay was estimated in four groups of chickens (10–90 samples in each group) identified based on the PCA results with tightly clustered populations considered as a single group. In Fig. 3, we show the LD decay plot of one large group (Group 4 = 90 samples) as representative of all populations (Table S1 and Fig. S1).

### Identification of genome-wide selective sweep signals

Since the geographical regions representing the studied populations showed little variation in annual mean temperature (Fig. 1C) but showed a large variation in annual rainfall (Fig. 1D), selection signature analyses (SSA) to uncover heat-stress adaptation were undertaken using two different approaches.

**Figure 4:**
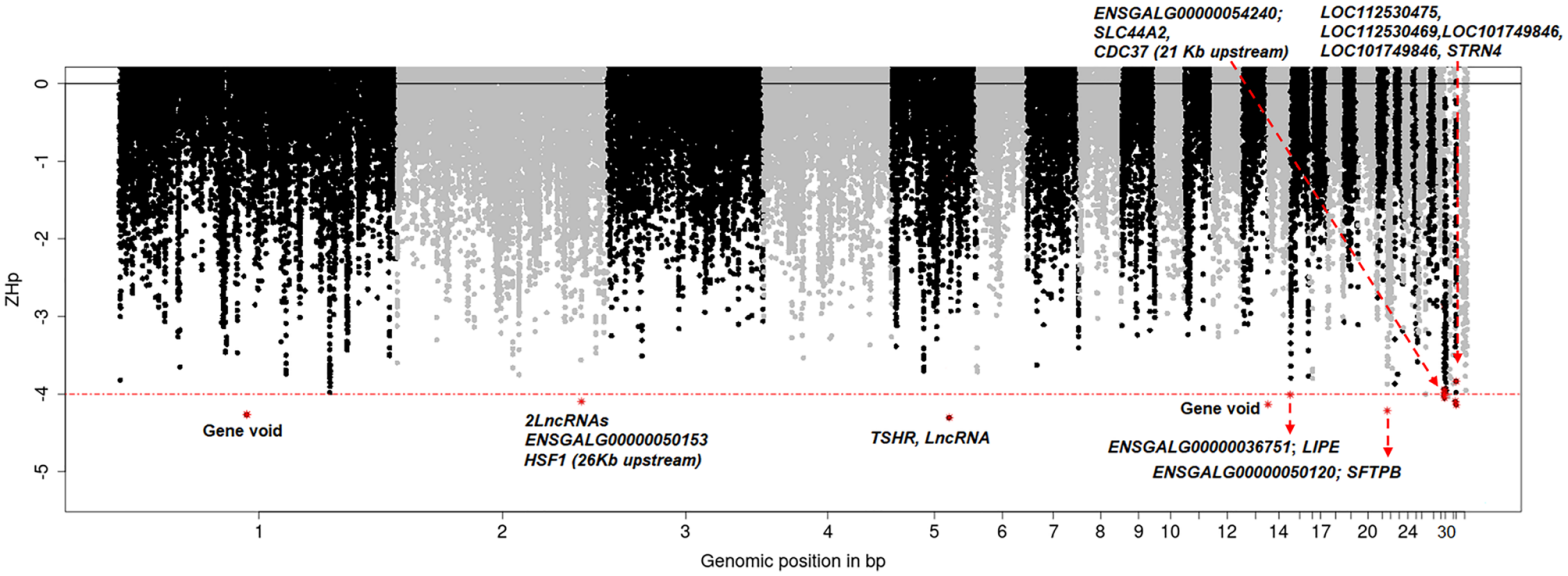
Manhattan plots from *Hp* analysis based on 12 hot climate populations.

In the first approach, the genomes of 12 of the 14 populations (omitting Wat-Karu and Dakan-Karu from the Mid-altitude region with lower temperatures) were combined as a single population. A within-population genomic search for low heterozygosity was performed using the “Pooled Heterozygosity” (*Hp*) approach described by Rubin et al.^12^ and was done in overlapping sliding windows of 20 kb size and a 10 kb step size. The goal of this analysis was to identify candidate genomic regions that are putatively under selection for heat-stress adaptation irrespective of other environmental conditions (e.g., arid or humid conditions or different agroecological conditions). The combined analysis of many populations allowed the reduction of spurious signals from any population structure and the detection of genomic regions with extremely low heterozygosity (Z*Hp* ≤ − 4) for the all hot-climate populations (Tables S2, S3 and Fig. 4, Fig. S2).

**Figure 5:**
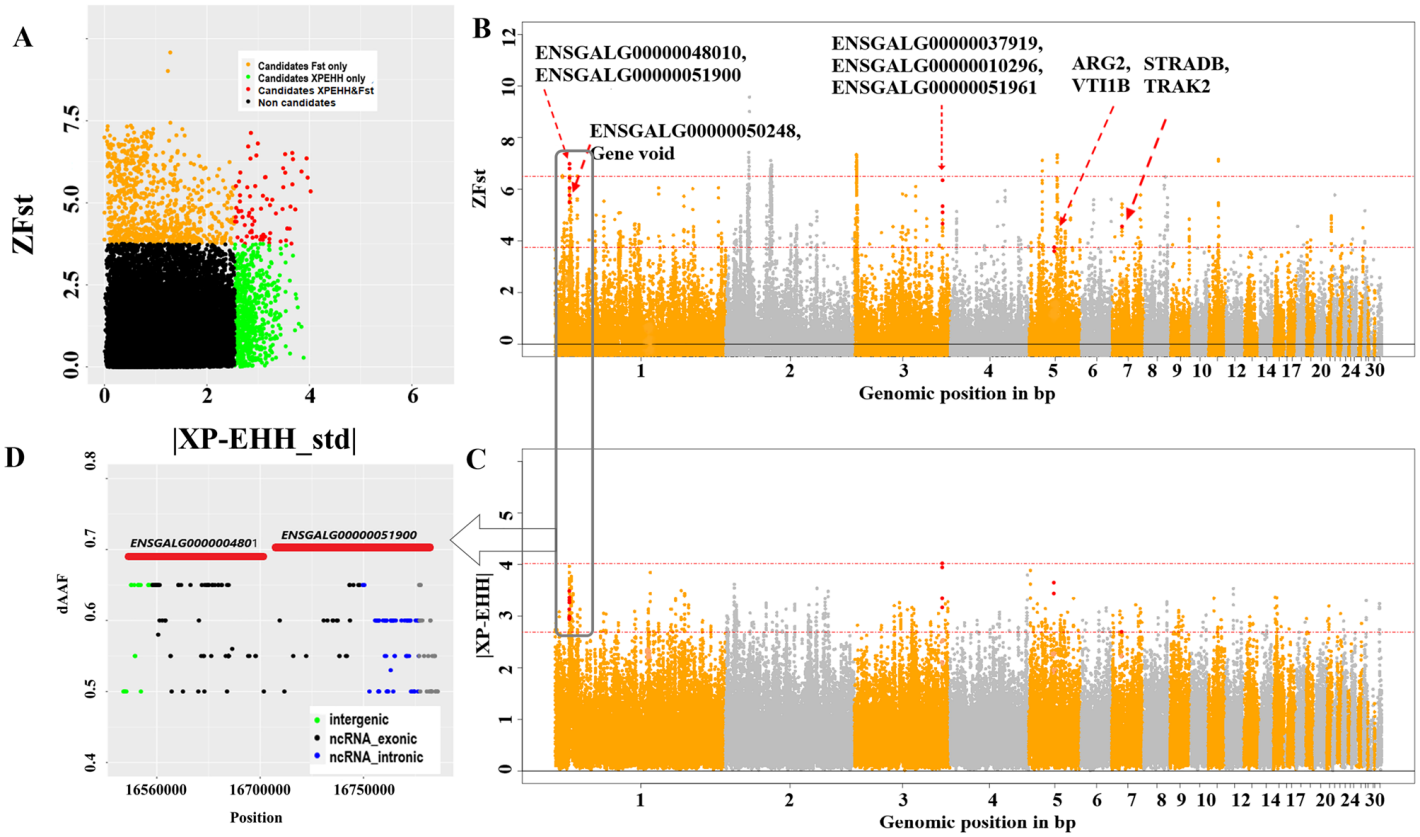
Selection signature analysis results for birds from regions of high and low precipitation. (**A**) Scatter plot of standardized values of *XP-EHH* versus *Fst*. (**B**,**C**) Manhattan plots for the *Fst* and *XP-EHH* analyses; common windows are marked with an asterisk along with gene names from common windows; the red dashed line represents *ZFst* and |*XP-EHH|* threshold. (**D**) Closer look at the common *Fst*/*XP-EHH* region—chr1: 16630000–16790000 with SNPs showing allele frequency difference (dAAF) > 0.5 between the hot arid and hot humid groups.

The second approach entailed a comparison of population groups from extreme hot-humid (Degema and Isiokpo) and extreme hot-arid (Jiga and Sabiyal) climates (Fig. 1D) to identify candidate regions that show large differentiation either in allele frequency spectrum (using *Fst* method) or LD pattern (using *XP-EHH* method) (Fig. 5). Again, two populations were combined in each extreme group to reduce any population structure effect and the analyses were performed using overlapping sliding windows of the same size as above. The rationale behind using two approaches for cross-group comparison was to gain more confidence in the results as these analyses had a much smaller sample size (10 samples per group) than the *Hp* approach used above. Candidate windows were generated from selection signature analysis with empirical P-value < 0.01, which had the thresholds as standardized *Fst* (Z*Fst*) > 3.7 or absolute standardized *XP-EHH* (|*XP-EHH*_std|) > 2.6 (Tables S2, S4, S5 and Fig. 5B,C, Fig. S3, S4). Only common windows or regions from both analyses were considered as putative selective sweeps (Tables S6, S7). *XP-EHH* analysis can indicate the directionality of selection. In our analysis, a positive *XP-EHH* value indicated selection in the hot-arid group while a negative value indicated selection in the hot-humid group (Table S6).

### Candidate loci and genes for heat-stress adaptation irrespective of the agro-ecologies

A total of 92,936 windows were analysed. Only 11 windows (0.02%) passed the genome-wide significance threshold of *ZHp* ≤ − 4 with mean of *Hp* = 0.022 (Fig. S2). These candidate selective sweep windows are located on chromosomes 1, 2, 5, 14, 22, 30, and 32 and overlap with 16 genes (except two windows which are gene-void) (Table 1 and Table S3). These overlapping genes—to be considered here as the candidate genes under positive selection—have highly relevant biological functions (Table 1) associated with thermotolerance, e.g., *TSHR*—has a role in thermogenesis (possibly regulated by an epigenetic mechanism, as fixed in most chickens^13,14^. *RYR1*-like genes (*LOC112530475* and *LOC101748756*)—involved in hyperthermia, *CYP450*-like genes (*LOC112530469* and *LOC101749846*)—role in oxidative stress response, *SFTPB*—role in respiratory gaseous exchange (affecting heat loss from the body), *LIPE*—lipid metabolism, *STRN4* and *SLC44A2*—roles in nervous system processes and immunity, and two long non-coding RNAs (lncRNAs) with possible cis-regulatory effect on the nearby Heat Shock Factor 1 (*HSF1*) gene^15–18^. The analysis of GO terms and KEGG pathways associated with these genes sheds further light on their potential relevance to heat stress adaptation (Table S8). For instance, *LIPE, LOC101749846,* and *LOC112530469* are involved in the glycerolipid catabolic process (GO:0046503) and organic acid metabolic process (GO:0006082), *LOC101749846* and *LOC112530469* are also associated with the molecular function GO term ‘oxidoreductase activity’ (GO:0016491), *TSHR* is involved in neuroactive ligand-receptor interaction (gga04080) and *LOC101748756* is involved in the calcium signalling pathway (gga04020) (Table S8).

**Table 1:** Candidate windows and genes under positive selection signatures in Nigerian indigenous chickens in relation to heat stress adaptation based on *Hp* analysis.

Sweep regions	Candidate genes and functions
Chr 2: 131020000–131040000	Contains multiple lncRNAs with possible cis-regulatory function on the nearby gene (*HSF1*) *HSF1* (Heat Shock Factor 1): 26 kb upstream, encodes a transcription factor that is rapidly induced after temperature stress and binds heat shock promoter elements^18,19^
Chr 5: 41000000–41020000	*TSHR*: Role in thermogenesis^20,21^
Chr 14: 16010000–16030000	*LIPE*: Hormone-sensitive lipase activity^22,23^
Chr 22: 40000–60000	*SFTPB*: Pulmonary surfactant protein; role in respiratory gaseous exchange^24,25^
Chr 30: 10000–30000	*SLC44A2*: Choline transport (important for the nervous system); involved in positive regulation of I-kappaB kinase^17,22,26^ Nearby gene: *ILF3* (16 kb downstream) has a role in chronic stress adaptation^27^
Chr 30: 200000–220000	*LOC107050992*: iron-sulpher binding and electron transfer activity^17,22^ Nearby gene: *CDC37* (21 kb upstream)^28,29^
Chr 32: 0–20000	*LOC112530475* and *LOC101748756*: both RYR1 like genes; RYR1 is involved in calcium channel activity and calmodulin-binding^17,22^
Chr 32: 590000–610000	*LOC112530469* and *LOC101749846*: Both are cytochrome P450 like genes; oxidoreductase activity and heme-binding^17,22^ *STRN4*: Calmodulin binding and calcium channel activities^22^ Nearby gene: *HIF3A* (16 kb downstream)^30,31^

### Candidate loci and genes for heat-stress adaptation in the hot-arid condition

Twenty-eight putative sweep regions were commonly detected by both *Fst* and *XP-EHH* analyses. The size of these regions ranged between 20 and 140 kb (average 35 kb), with a combined total length of 1100 kb and an average length of 35 kb. These regions are distributed across chromosomes 1, 2, 3, 5, 6, 7, 8, 11, and 19 (Tables S6, S7). Chromosome 1 has the longest length of selection signature region, and chromosomes 11 and 19 the shortest length (20 kb) compared to other regions. A total of 34 genes are found within these regions including 10 long non-coding RNA (lncRNA), and 21 protein-coding genes, with five regions being gene deserts (Table 2, Table S6, Fig. 5B). Table 2 shows that the overlapped genes are involved in cytokine activities, inflammatory responses, and immune responses (e.g., *TAFA5, TRIM24, AGR2, CHID1, ARG2 CKLF, CLTM3, SUPT4H1*) and include a sub-set of protein-coding genes with highly relevant stress response functions. Those genes also showed certain GO terms, pathways, and QTLs that relate to disease resistance and immune responses (Table S9, Figs. S5, S6). Other gene functions include those of the nervous system (*DPY19L1* and *BICD1*), calcium ion transport (*TSPAN13*), abdominal fat deposition (*KALRN*) and bone formation (*MRPS18*).

**Table 2:** Candidate genes under positive selection signatures in hot-arid conditions.

Sweep regions	Candidate genes and functions
Chr1: 17600000–17680000	*TAFA5:* role in cytokine activity^22^
Chr1: 56420000–56440000	*TRIM24:* involved in cytokine pathways and the inflammatory response^32,33^
Chr1: 59080000–59110000	*BICD1:* participates in the development and function of the nervous system^34^
Chr2: 28560000–28580000	*TSPAN13:* involved in the regulation of calcium ion transmembrane transport^22^ *AGR2:* involved in the inflammatory response^22^
Chr2: 47230000–47260000	*DPY19L1:* role in neuronal migration in the developing mouse cerebral cortex^35^
Chr3: 30900000–30920000	*MRPS18A:* cortical bone formation^36^
Chr5: 15250000–15290000	*CHID1:* involved in negative regulation of cytokine production; inflammatory response^22^
Chr5: 15320000–15380000	*MUC6:* involved in the maintenance of gastrointestinal epithelium; intestinal barrier function in chicken^22,37^
Chr5: 29080000–29100000	*ARG2*: anti-inflammation associated^38^
Chr7: 27830000–27850000	*KALRN:* key regulatory role in abdominal fat deposition^39^
Chr7: 32950000–32980000	*ARHGAP15:* involved in signal transduction^22^
Chr8: 27480000–27510000	*NFIA:* necessary for articular cartilage differentiation^40^
Chr11: 11440000–11460000	*CMTM3:* involved in positive regulation of B cell receptor signalling pathway^22,41^ *CKLF:* this may play an important role in the inflammation and regeneration of skeletal muscle^42^ *TK2:* kinase activity^22^
Chr19: 710000–730000	*SUPT4H1:* viral infection pathway^43^

### Candidate loci and genes for heat-stress adaptation in the hot-humid condition

Only three common selective sweep regions (i.e., regions detected by both *Fst* and *XP-EHH* analysis as candidates) were detected for the hot-humid climate. These are located on chromosomes 1, 5 and 7 (all are 20 kb in size) and overlap with six genes—comprising two lncRNAs and four protein-coding genes (Table 3, Tables S6 and S7) involved in immune response (*ARG2, VTI1B* and *STRADB)* and intracellular transport (*TRAK2*). A summary of the molecular functions, biological processes, and pathways is presented in Table S10 and Figs. S7 and S8.

**Table 3:** Protein coding genes overlapping with putative selection signatures in populations from hot-humid conditions.

Sweep regions	Relevant biological functions for the candidate genes
Chr5: 29080000–29100000	*ARG2:* mitochondrial type of arginase that leads to an increase in oxygen-free radical formation and endothelial dysfunction^44^, arginine metabolism is also a critical regulator of innate and adaptive immune responses^45^
Chr7: 11480000–11500000	*VTI1B:* concerned with increased secretion of cytokines associated with cellular senescence^16^ *STRADB*: among its related pathways are MTOR signalling and AMP-activated protein kinase signalling^22^ *TRAK2*: Predicted to be involved in several processes, e.g., mitochondrion distribution; organelle transport along the microtubule and protein targeting^22^

## Discussion

This is the first study performing a large-scale WGS analysis on NICs to assess genetic diversity and identify genomic signatures of adaptive selection in relation to hot (humid or arid) climates. The study has high coverage of chicken populations representing the diversity of the Nigerian landscape, providing the opportunity to investigate both within and between population genetic diversity, as well as adaptive diversity. Our study generates and utilizes a powerful and robust variant dataset by jointly analysing a large number of samples (n = 120) using established bioinformatic workflow of GATK. Such joint analysis is known to improve the sensitivity and accuracy of the detection greatly^46^. Therefore, the variant dataset along with the WGS data contributes major genomic resources for further research on chicken.

By comparing SNPs from NICs and those available in public databases as well as those recently detected in Ethiopian indigenous chickens by our group^47^, we have detected ~ 11% novel chicken variants. Our study reveals large within-population genetic diversity in NICs, but a low level of genetic differentiation between populations. This result corroborates the findings of previous diversity studies based on mitochondrial DNA (mtDNA) D-loop in NICs, which reported that, currently, all the sequences belong to a single clade or haplogroup, predominantly found in South Asia (Indian subcontinent). It supports a single geographic origin in Asia (Indian subcontinent) and suggest a extensive genetic intermixing within the country, thus resulting in a lack of mtDNA phylogeographic structure among the NICs^10,48^.

The value and pattern of LD decay detected in the present study is similar to that observed in a previous study of Korean native chicken^49^, and some Chinese indigenous chicken^50^ (Wenchang, Beijing You, Taihe Silkies, and Shouguang). They showed a rapid decay in LD structure that is generally common in local breeds or populations that experienced less intensive breeding programs compared to commercial chicken breeds^51^_._ Genomic LD structure can be affected by various factors including effective population size, non-random mating, admixture, genetic drift, selection, mutation, and recombination rate. The rate of LD decay can therefore be used to measure the evolutionary history of populations^52^ and are also helpful for determining the resolution of association mapping or assessing the desired number of SNPs to be used for genome-wide association analysis.

**Figure 6:**
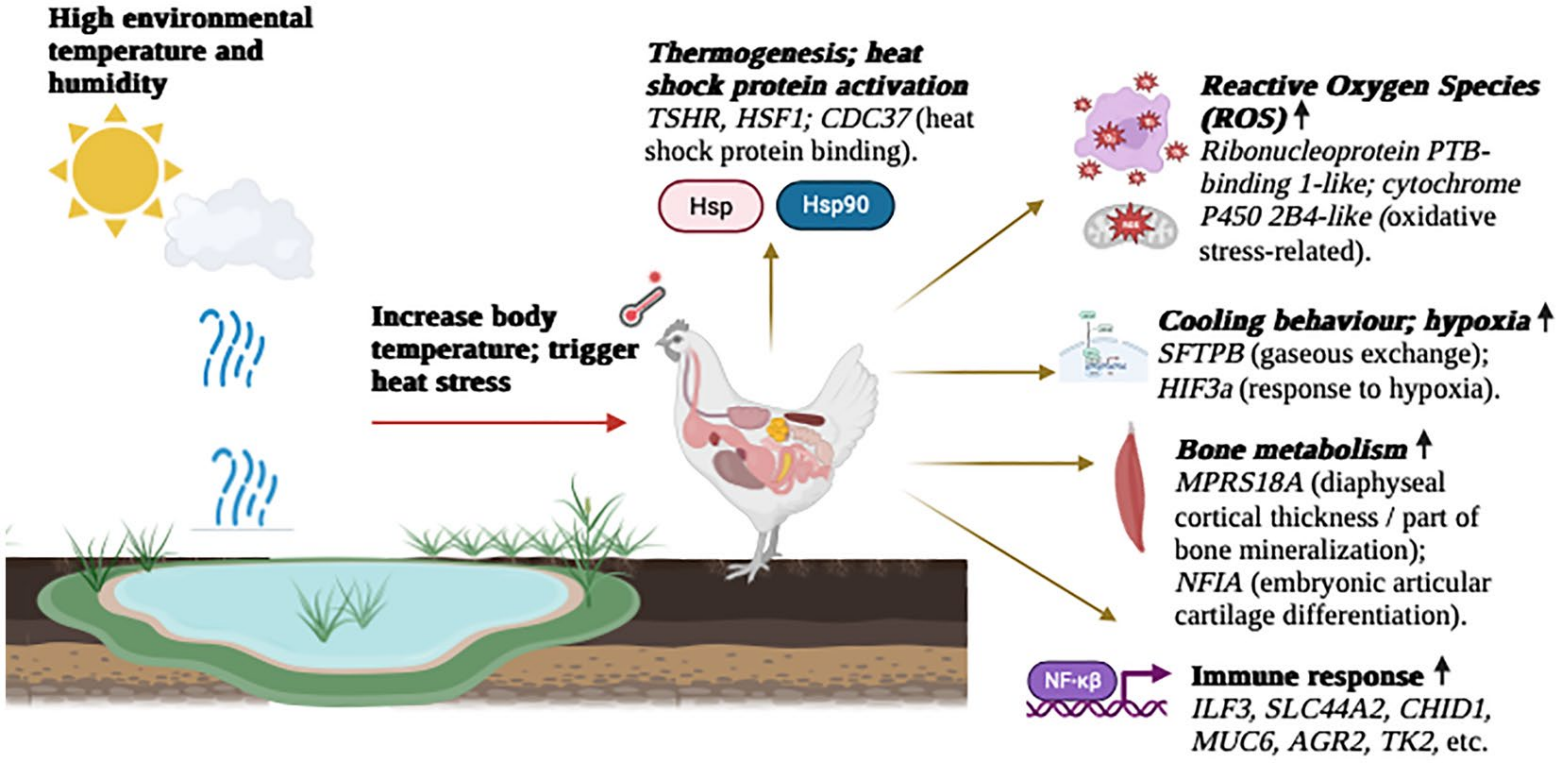
Summary of the main effects of heat stress on Nigerian indigenous chickens. (Created with BioRender.com).

A major focus of our study was to identify candidate genes and pathways related to thermotolerance in the NICs. Important candidate genes and their involvement in thermoregulation can be summarized schematically as Fig. 6. Chicken activates thermoregulation mechanisms to lose heat when the environmental temperature is above the thermoneutral zone by showing three types of responses: behavioural, biochemical, and physiological. It is also notable that adaptive response to heat stress occurs not only with high temperatures but is also affected by the relative humidity of the environment.

The *TSHR* gene, detected in the *Hp* analysis, overlapped with the strongest peak (*ZHp* = − 4.3) and is considered here as a major candidate gene for tropical heat-stress adaptation irrespective of arid or humid conditions. Many studies have demonstrated that *TSHR* may be involved in reproduction, regulating energy balance, metabolism, and thermoregulation^53,54^. Moreover, recent studies found that it functionally contributed to the chicken response and adaptation to hot and tropical environments^21,54^. This gene also has been detected in selection signature analyses from most domestic chicken populations^12,55,56^. The potential role of epigenetic regulation of thermotolerance in chicken has also been expressed by Karlsson et al.^57^ and Gheyas et al.^47^ The study from Guo et al.^21^ reported that a missense mutation in the *TSHR* gene might regulate the metabolic rate to enhance heat tolerance and contribute to chicken adaptation to high ambient temperature in tropical climates.

In this study, several lncRNAs were found to overlap with putative sweep regions. LncRNAs have potential regulatory functions on gene expression exerted in either a cis-acting or trans-acting manner on their target genes. Cis-acting lncRNAs regulate the expression of target genes that are located at or near the same genomic locus while trans-acting lncRNAs either inhibit or activate gene transcription at independent chromosomal loci^58^. In chickens, lncRNAs have been reported to regulate muscle development, lipid metabolism, egg production and disease resistance^59,60^. In our study, the heat shock transcription factor 1 (*HSF1*) gene, is found 26 kb upstream of a candidate region on chromosome 2 which overlaps with three lncRNAs. This gene functions as a stress-inducible and DNA-binding transcription factor that plays a role in the transcriptional activation of heat shock response (HSR) leading to the expression of a large class of molecular chaperones of heat shock proteins (HSPs) that protect cells from cellular damage in the chicken^18,61^. Consequently, *HSF1* is associated with several gene ontology terms like ‘cellular response to heat (GO:0034605)’ and ‘heat shock protein binding (GO:0031072)’^17^ and has been proposed as a marker during acute heat stress in chickens^61^. Another gene, *CDC37* (Cell Division Cycle 37) which is located 21 kb upstream of another candidate region on chromosome 30 also overlaps with a lncRNA. *CDC37* has the molecular function of ‘heat shock protein binding (GO:0031072)’ and probably acts as a co-chaperone of *HSP90* or non-client protein binding partner that also assists in repairing denatured proteins or promoting their degradation caused by heat stress^29^.

Previous studies have explored the links between putative novel lncRNAs and previously reported QTLs; for instance, a novel lncRNA (*LncFAM*) was found located in a chicken growth QTL^62^, while another study revealed the association of certain lncRNAs with response to Marek’s Disease Virus (MDV) in commercial egg production lines^63^. In our study, we found regions from *Hp* and shared *Fst*-*XPEHH* analyses that overlapped with both lncRNAs and QTLs (related to immune response, fear behaviour, and Mareks’s disease susceptibility (Table S6). However, the role and function of these lncRNAs with nearby protein-coding genes and the overlapping QTLs in relation to heat stress adaptation still requires further investigation.

Various kinds of stress, including extreme environmental temperatures, lead to the generation of reactive oxygen species (ROS), causing oxidative stress and lipid peroxidation. Through *Hp* analysis, we have found several candidate genes on chromosomes 30 and 32 (*LOC107050992* (ribonucleoprotein PTB-binding 1-like), and cytochrome P450 2B4-like*: LOC112530469* and *LOC101749846*) which are associated with oxidative stress. These genes are involved in several GO terms^17^ associated with oxidoreductase activity (GO:0016491) and oxidation–reduction process (GO:0055114). Oxidative stress from heat exposure can manifest in all parts of the body, but mitochondrial dysfunction is central to oxidative stress. In the initial acute heat stress phase, mitochondrial substrate oxidation and electron transport chain activity are increased, resulting in excessive superoxide (a type of ROS) production^64^. During gaseous exchange in heat stress at high ambient temperature, a bird’s respiratory rate is enhanced to dissipate heat. From the *Hp* analysis (of combined population) we found a sweep region on chromosome 22 that overlaps with the Surfactant Protein B (*SFTPB*) gene which is involved in the biological process of gaseous exchange between an organism and its environment (GO:0007585). The respiratory system of birds exposed to heat stress operates both for gaseous exchange and as the evaporative cooling system^65^.

A study by Varasteh et al.^66^ reported that chicken’s critical adaptive response to heat stress increases the peripheral blood flow, resulting in reduced blood supply in the intestines and a hypoxia-induced oxidative stress response. A sweep region detected on chromosome 32 from the *Hp* analysis overlaps with gene *LOC101749846* and is nearby to *HIF3a* (Hypoxia-inducible factor 3 subunit alpha), which is involved in the epoxygenase P450 pathway (GO:0019373), response to hypoxia (GO:0001666) as well as angiogenesis (GO:0001525) in chicken^17^. This finding is in line with the study from Zahoor et al.^67^ who reported angiogenic pathways are involved in hypoxia-induced angiogenesis in chickens.

One of the most noticeable developmental problems associated with heat stress in poultry is a pronounced induction of leg abnormalities, as shown in broilers, layers, and turkeys^68^. Elevated temperatures impair gut integrity, thereby increasing systemic inflammation that elicits osteoclastic bone resorption that is related to bone metabolism such as cortical thickness in diaphysis in tibia bone as suggested by Zhang et al.^68^. The *MRPS18A* gene, which overlaps with a sweep region from the arid region population was found to be a candidate for the diaphyseal cortical thickness (part of bone mineralization) that is associated with heat shock factors^36^ which are involved in bone formation. *MPRS18A* encodes a mitochondrial ribosomal protein. Since mitochondrial function is crucial for cellular metabolism, such mutation can therefore affect the functions of diverse organ and tissue systems, including bone and active osteoclasts that are rich in mitochondria^36^.

Immunity is suppressed under heat stress conditions as has been observed in previous studies^69,70^. To protect the body from the adverse effects of heat stress, a defence mechanism is activated in chickens. Initially, the early response system stimulates the central nervous system, and eventually, the immune system is involved. Based on *Hp* and shared *Fst-*XP-EEH regions we have found two genes (*SLC44A2* and *BICD1*, respectively) which participate in the function of the nervous system^22,34^. These genes are potentially involved in immunity as the central nervous system modulates immune responses, which are mediated by a complex network of signals. These signals interplay across the nervous, endocrine, and immune systems which affect metabolism and immune responses^71^. Many immunity-related genes have been detected in all three selection signature analyses. Some of the genes located nearby to the sweep regions based on *Hp* analysis are immune-related genes that might have cis-acting or trans-acting interaction with their target genes. These include Interleukin Enhancer Binding Factor 3 (*ILF3*), and Solute Carrier Family 44 Member 2 (*SLC44A2*)—both contributing to the negative regulation of viral genome replication (GO:0045071) and are involved in innate immune system pathways and positive regulation of I-kappa B kinase/IκKβ (GO:0043123). Several genes related to immune response have also been found in the common sweeps from *Fst* and *XP-EHH* analyses, for instance, the cytokine-related genes: *TAFA5, TRIM24, CHID1, MUC6* (from arid climate)*; VTI1B* (from humid climate); infection and inflammation-related genes: *AGR2, TK2, CMTM3, SUPT4H1* (arid climate); *SETD4, ARG2, VTI1B* and *STRADB* (humid climate). Heat stress significantly impacts the immunity and cytokine expression of chickens. Heat stress was found to modulate the gene expression of a range of different cytokines in chickens and many studies have demonstrated that bacteria are exploiting neuroendocrine alterations following stress response in the host to promote growth and pathogenicity.

## Conclusions

This study has generated and characterized over 17 million high-quality genome-wide SNPs, of which ~ 11% are novel variants. These large numbers of SNPs provide an additional resource for future applications and characterization of NICs. The identification of candidate genes/genomic regions under selection will help in understanding their evolution and functional roles in relation to environmental challenges. The small number of highly plausible candidate genes detected for hot climate adaptation, are seen to be involved in relevant biological processes and pathways related to oxidative stress (e.g., *cytochrome P450 2B4-like: LOC112530469* and *LOC101749846, SFTPB*), cellular responses to heat and hypoxia (e.g., *HSF1*, *CDC37*, *HIF3A*), transcriptional regulation (*TSHR*), immune response (*SLC44A2, ILF3*), and metabolic activities—all of which are important for thermal adaptation. This study also enhances our understanding of the role of natural selection in shaping the genome of NICs for adaptation to both hot-arid and hot-humid tropical conditions. Apart from the genetic adaptation, this study dissects the within and between-population genetic diversity in Nigerian indigenous chicken populations. Understanding genetic diversity is a prerequisite in setting up an effective breeding program and selecting the population to use. Our study shows that there is large genetic diversity within Nigerian chicken populations that can be harnessed for breeding improvement of locally adapted birds.

Taken together, these findings will help guide the improvement of indigenous chickens by helping design specific breeding programs as well as poultry management strategies to minimise heat stress and enhance disease resistance and productive performance. This will increase the contribution of poultry products to global food security.

## Materials and methods

### Chicken sampling

Sampling was performed to represent diverse Nigerian agro-climatic zones. From each zone, two villages were selected and from each village 4–10 scavenging chickens were sampled by drawing blood (50–250 μl) from the wing vein with the logistical support and agreement of the Department of Animal Sciences, Obafemi Awolowo University (Ife Ife, Nigeria). All animal works were approved by the Institutional Animal Care and Use Committee of the International Livestock Research Institute (IREC2017-26) and were handled strictly in compliance with the guidelines of this committee. A geographic coordinate (latitude and longitude) was collected for each sampled village for the extraction of environmental data from public databases.

All the collected blood samples were processed for DNA using the Qiagen DNeasy blood and tissue kit protocol (https://www.qiagen.com/ca/resources/download.aspx?id=63e22fd7-6eed-4bcb-8097-7ec77bcd4de6&lang=en). The genomic DNA (gDNA) from each sample was then normalized to a final volume of 100 µl and final concentration of 50 ng/µl and was sent to Edinburgh Genomics (http://genomics.ed.ac.uk/) in the UK for whole-genome sequencing.

### Sequencing and variant calling

Whole genome sequencing was performed on the Illumina HiSeqX platform, with an average 64 × paired-end coverage. Sequence reads were mapped against the chicken reference genome (GRCg6a) (https://www.ncbi.nlm.nih.gov/datasets/genome/GCA_000002315.4/) using the BWA-mem algorithm^72^. Variant calling, filtration, and genotyping were performed by combining all 120 samples together following the best practice protocol of the GATK package for “Germline short variant discovery”^73^, involving the Haplotype Caller method and Joint Genotyping of all samples together. Initial filtration on SNP calling was performed using the GATK’s machine learning algorithm, the VQSR (Variant Calling Score Recalibration) approach for which 1 M validated chicken SNPs were used as a ‘training’ and ‘true’ set and ~ 20 M publicly available SNPs from the Ensembl database was used as a ‘known’ set. Further filtration on SNPs was applied before using for genomic analysis as: biallelic SNPs, minor allele frequency > 0.05, genotype quality > 15.0, depth of coverage > 3, maximum missing genotype rate < 20% and Hardy–Weinberg-Equilibrium (HWE) probability < 1 × 10^−7^. Only autosomal SNPs were used for genetic diversity and selection signature analyses (SSA). Quality checks for samples were performed based on genotype missing rate and relatedness analysis in VCFtools v0.1.15^74^. Thus an individual pair showing higher than expected relatedness (> 0.9) was removed. For downstream analyses, variants were extracted for individual populations using option “*gatk SelectVariants*” from GATK (https://gatk.broadinstitute.org/hc/en-us/articles/13832694334235-SelectVariants).

### Genetic diversity analysis

Inbreeding coefficient for individual chickens, nucleotide diversity (π or “pi”) for the individual chicken population, and pairwise population-differentiation (*Fst*) between populations were calculated using the filtered SNP variant set in VCFtools (v0.1.15). The average genome nucleotide diversity and *Fst* were estimated in 20 kb windows with 10 kb sliding steps. Population structure among the investigated populations was inferred with PCA using ‘smartpca’ in ‘eigenstrat’ version 6.0.1^75^. The proportion of ancestry (admixture) in each individual and population was estimated using ADMIXTURE version 1.3.0^76^ considering K values from 1 to 5 with the lowest cross-validation error used to choose the best K value. The PCA analysis was performed with an LD-pruned set of SNPs consisting of about 4 M variants. LD pruning was performed in Plink (v1.9) (https://www.cog-genomics.org/plink/1.9/ld) with the options “*--indep-pairwise 50 5 0.5*”. For admixture analysis, a 30% thinning of the SNPs was performed after the LD pruning to reduce the computation burden (retaining 583,700 SNPs). A pairwise *r*^2^ estimation was used to measure LD between pairs of SNPs within a chromosome using the PopLDdecay (v3.40) program^77^. SNPs on both autosomal and sex chromosomes that passed the quality control using options “-MAF 0.05” (minimum minor allele frequency of 0.05) and “-MaxDist 15” (maximum window bin 15 kb) were used. The decay of LD was plotted using the” ggplot” package (https://ggplot2-tidyverse.org) in Rstudio version 3.4.3.

### Selection signature analyses

Selection signature analysis (SSA) was performed using Pooled Heterozygosity (*Hp*)^12^, *Fst*^78^ and *XP-EHH*^79^ approaches in overlapping sliding windows (20 kb size with 10 kb step) with at least 10 SNPs/window from the combination of multiple populations (Table S2). The weighted *Fst* values were standardized (Z*Fst*) to allow the setting of the same threshold across analyses. *XP-EHH* analyses were carried out using the Hapbin package^80^ after removing SNPs with missing genotypes. *XP-EHH* analyses were first performed for individual SNPs and then mean values were calculated within windows for both the standardized *XP-EHH* (*XP-EHH*_std) and the absolute value of *XP-EHH*_std. SSA windows with empirical P-value < 0.01 were considered as putative selective sweeps for a standardized *Fst* (Z*Fst*) > 5 or an absolute standardized *XP-EHH* (|*XP-EHH*_std|) > 3.5. Moreover, since the positive and negative values of *XP-EHH* indicate the directionality of selection, all SNPs within an *XP-EHH*-based candidate window are needed to show the same directionality.

### Function analysis of candidate gene and SNPs

Bedtools version 2.25.0^81^ was used to merge the overlapping selected windows. Chicken genes that overlapped genomic windows passing the significant selective sweep threshold were retrieved from the Ensembl Genes 106 database using the Biomart online tool (http://www.ensembl.org/biomart). The candidate genes were then processed in a web-based PANTHER Classification System^82^ and KEGG Pathway Database (https://www.genome.jp/kegg/pathway.html) to map the candidate genes to known biological processes, molecular function, cellular processes, and molecular pathways. Candidate genes were also checked for their overlap with known chicken QTLs (ChickenQTLdb: https://www.animalgenome.org/cgi-bin/QTLdb/GG/index).

### Ethics statement

All animal works were reviewed and approved by the Institutional Animal Care and Use Committee of the International Livestock Research Institute (IREC2017-26) and were handled strictly in compliance with the guidelines of this committee. Written informed consent was obtained from the owners for the participation of their animals in this study.

### Supplementary Information

Supplementary Figures.

Supplementary Tables.

## Data Availability

The whole genome sequence data used in this study have been deposited in the European Nucleotide Archive (ENA) (https://www.ebi.ac.uk/ena) under study accession number PRJEB39536.
