# Supplementary material for: Genomic analysis of Nigerian indigenous chickens reveals their genetic diversity and adaptation to heat-stress
Source: Sci Rep. 2024 Jan 26;14:2209. doi: 10.1038/s41598-024-52569-4 (PMC10817956; doi:10.1038/s41598-024-52569-4)
Supplement: Supplementary file 1 — Supplementary Figures. [file 41598_2024_52569_MOESM1_ESM.docx]

**Genomic analysis of Nigerian indigenous chickens reveals their genetic diversity and adaptation to heat-stress**

**Mifta P. Rachman^1*^, Oladeji Bamidele^2^, Tadelle Dessie^3^, Jacqueline Smith^4^, Olivier Hanotte^3,5*^, Almas A. Gheyas^4*^**

^1^School of Biosciences, University of Nottingham, LE12 5RD, United Kingdom; ^2^African Chicken Genetic Gains (ACGG), Department of Animal Sciences, Obafemi Awolowo University, Ile Ife 220282, Nigeria;

^3^LiveGene – CTLGH, International Livestock Research Institute (ILRI), P.O. Box 5689, Addis Ababa, Ethiopia;

^4^Centre for Tropical Livestock Genetics and Health (CTLGH), Roslin Institute, University of Edinburgh, EH25 9RG, Edinburgh, United Kingdom;

^5^School of Life Sciences, University of Nottingham, NG7 2RD, Nottingham, United Kingdom.

^*^Correspondence: Mifta P. Rachman, email: mmiftarachman@gmail.com; Olivier Hanotte, email: o.hanotte@cgiar.org; Almas A. Gheyas, email: almas.gheyas@stir.ac.uk.

Figure S1. LD decay plot for all groups.


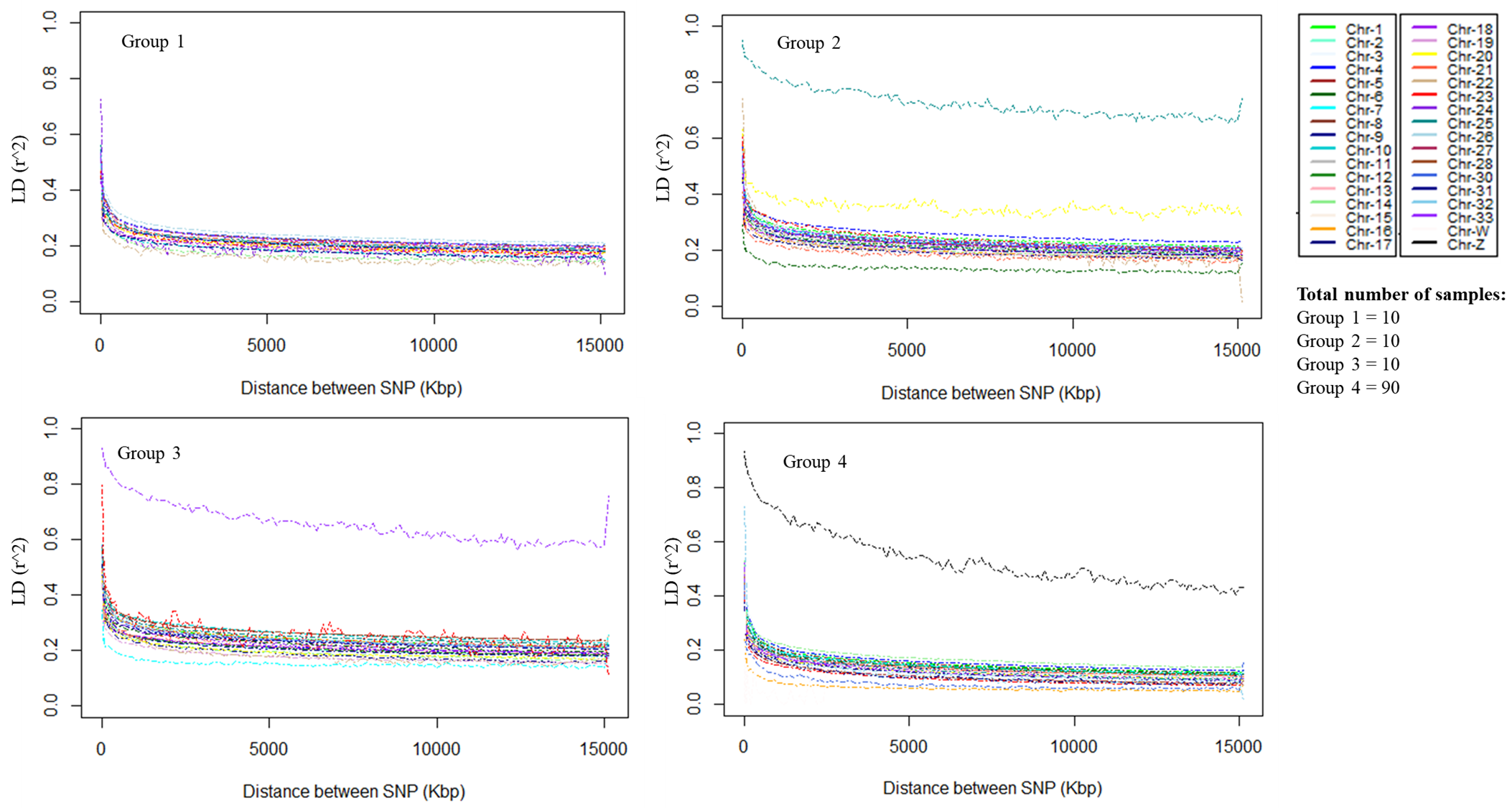


Figure S2. Histogram of Hp/ZHp analysis.


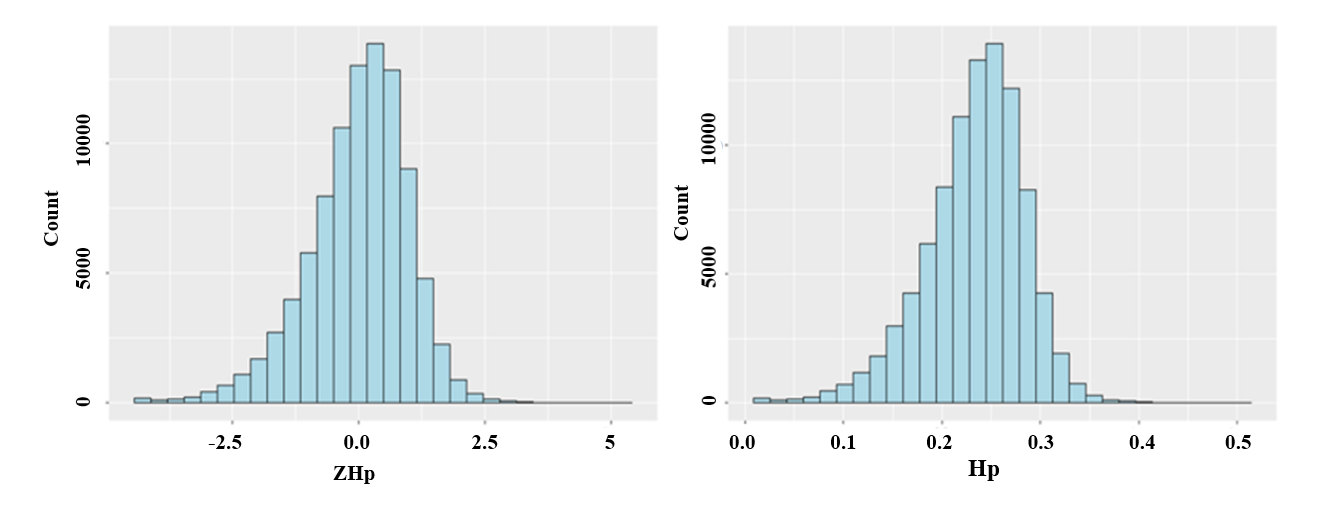


Figure S3. Histogram of Fst/ZFst analysis.


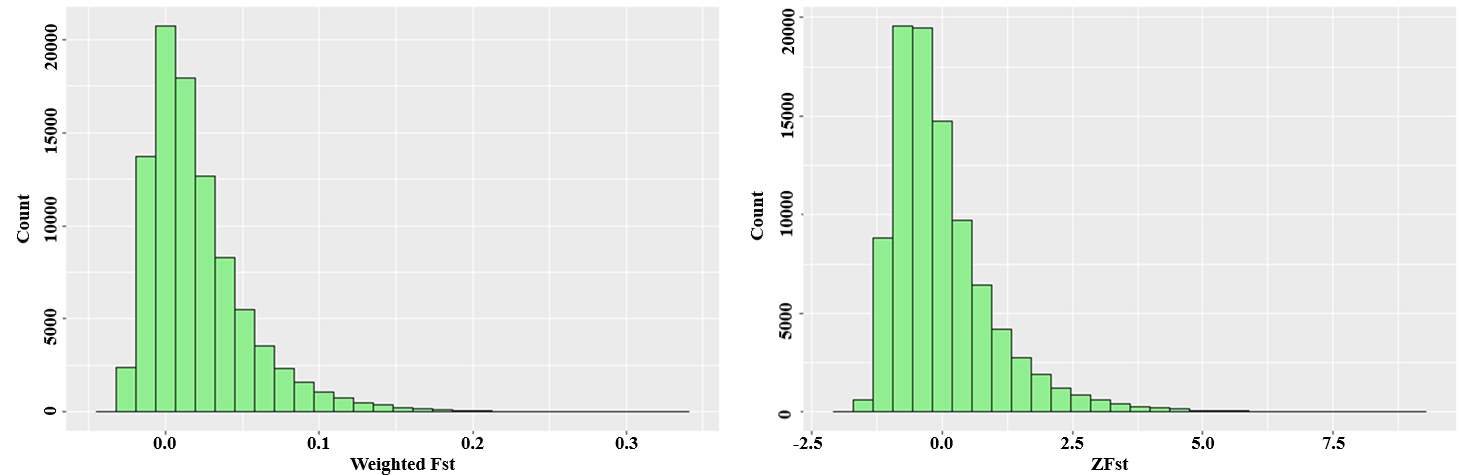


Figure S4. Histogram of XP-EHH analysis.


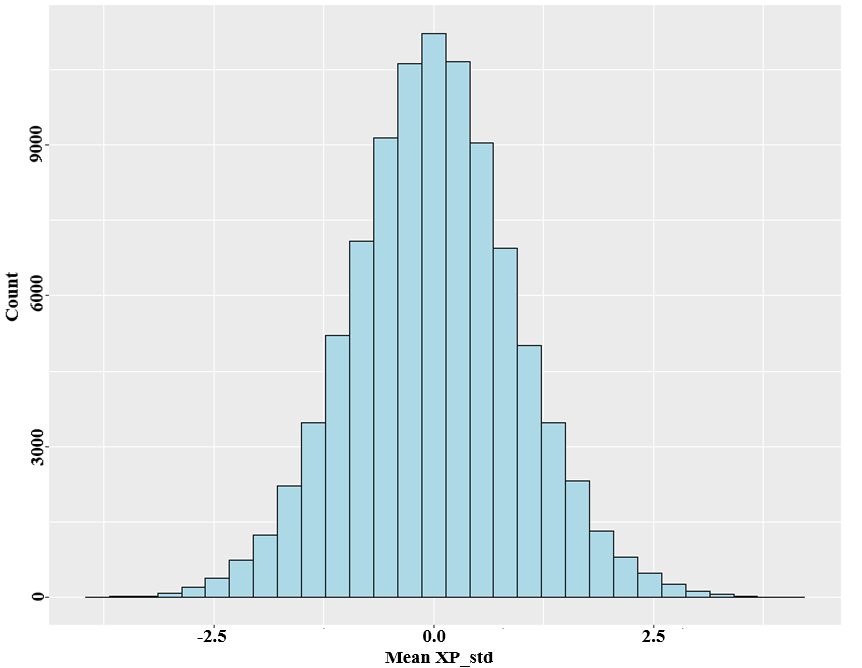


Figure S5. Summary GO and pathways in hot-arid region.


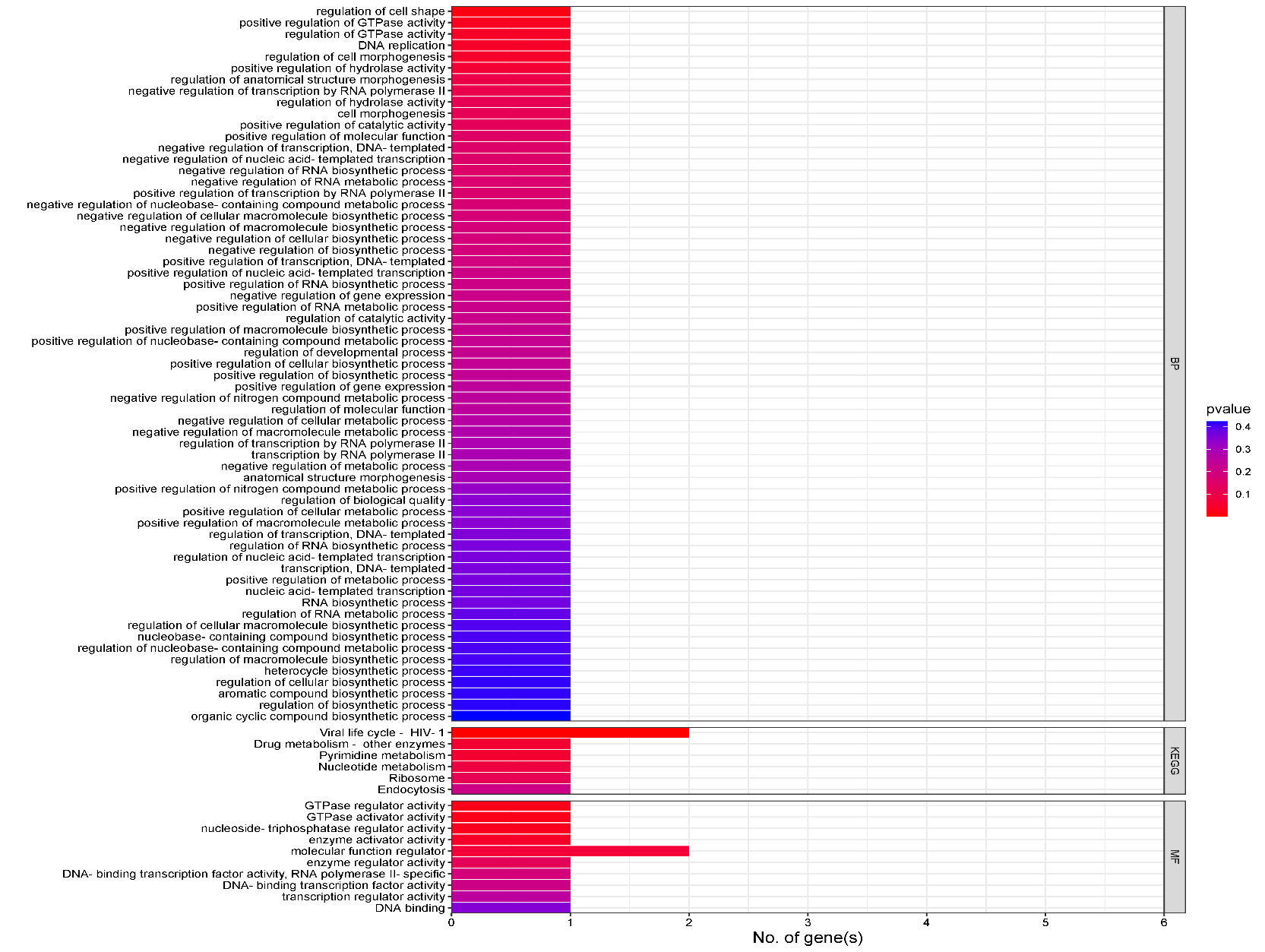


Heatmap was plotted by http://www.bioinformatics.com.cn/srplot.

Figure S6. GO and Pathway Enrichment score in hot-arid region.


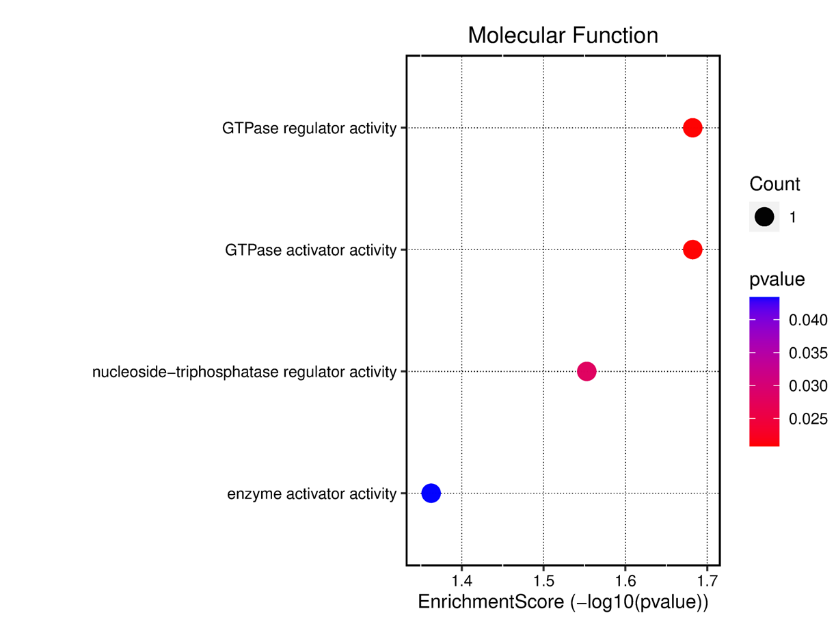

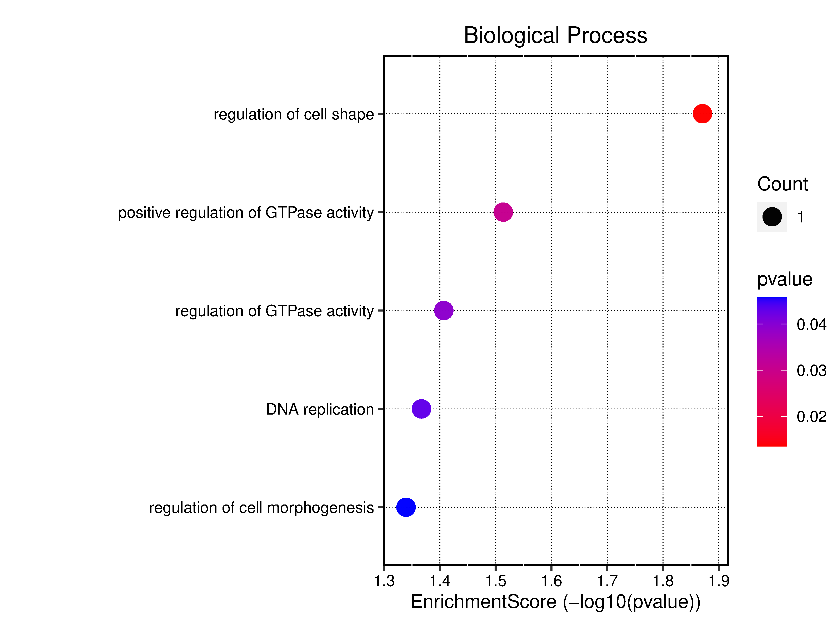

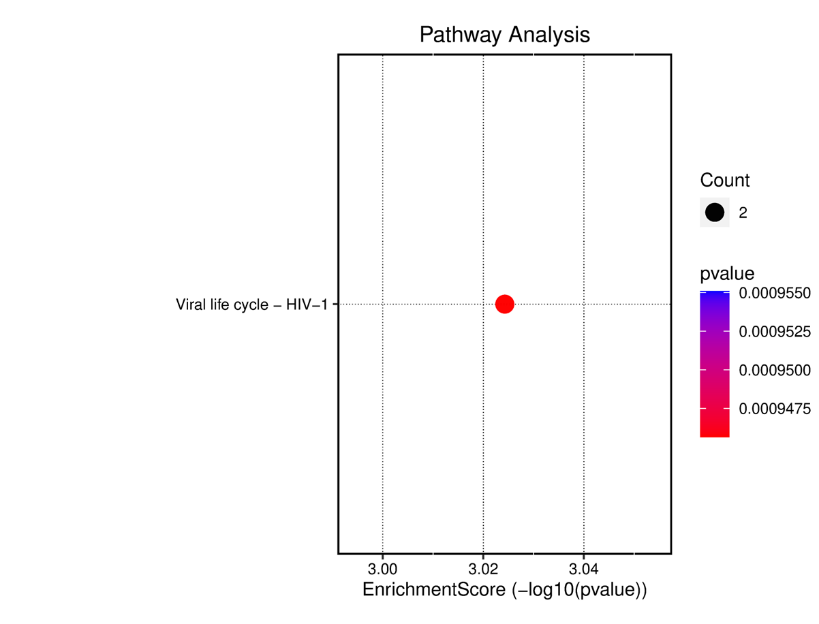


Heatmap was plotted by http://www.bioinformatics.com.cn/srplot.

Figure S7. Summary GO and pathways in the hot-humid region.


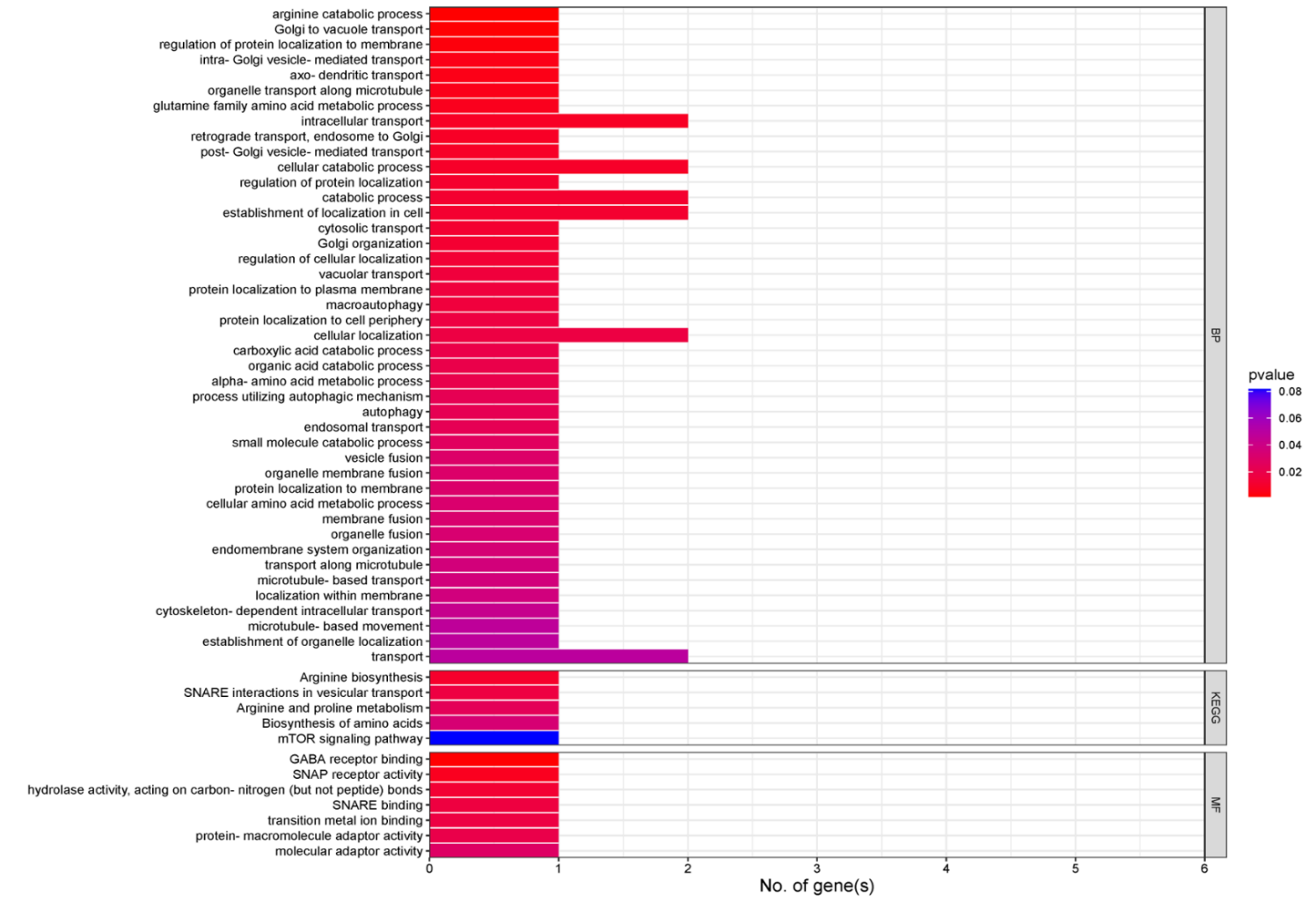


Heatmap was plotted by http://www.bioinformatics.com.cn/srplot.

Figure S8. Pathway enrichment score in hot-humid region.


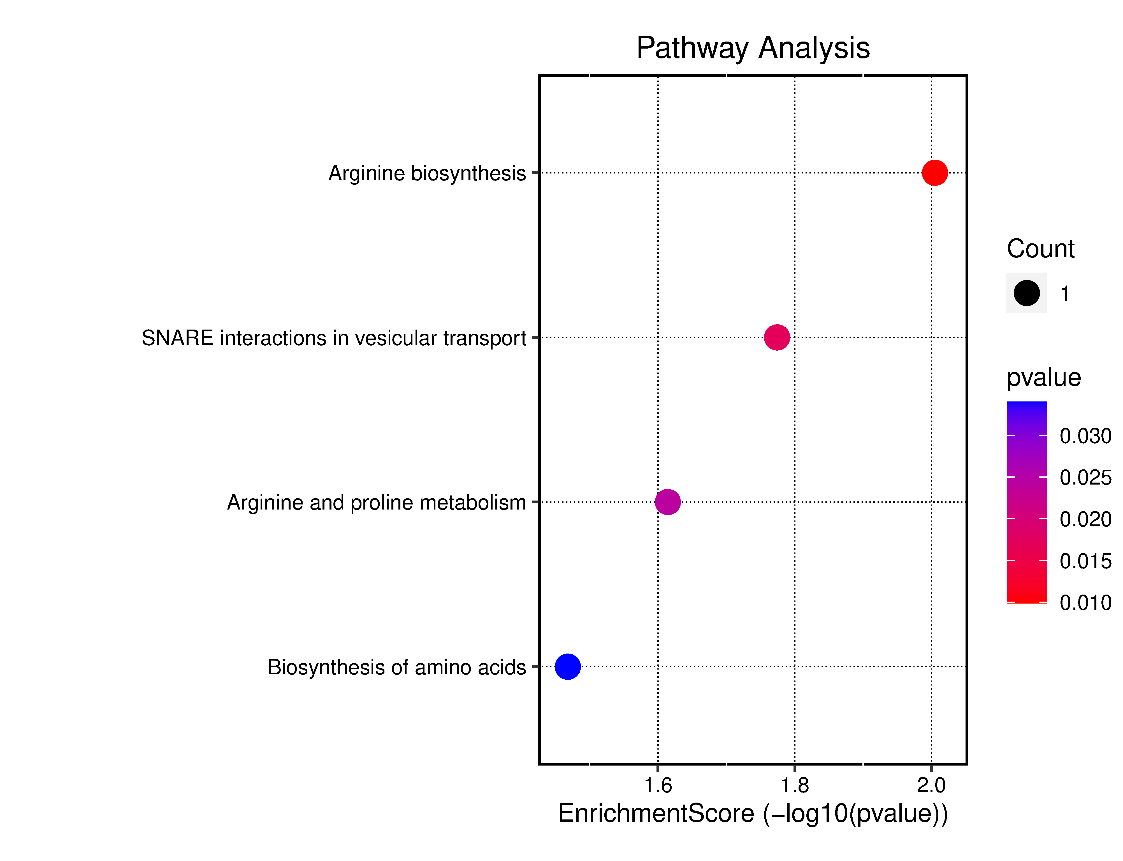


Heatmap was plotted by http://www.bioinformatics.com.cn/srplot.
